# Rubber Work

**Published:** 1861-02

**Authors:** Geo. L. Paine

**Affiliations:** Xenia, O.


### RUBBER WORK.

(Read at the January meeting of Mad River Valley Dental Society.)

BY GEO. L. PAINE, D. D. S., XENIA, O.

In accordance with an appointment made by this Society, at its last meeting, I proceed to discharge the duty imposed on me of reading an essay on "Vulcanite Work." I regret that this duty was not imposed on some other member, better qualified, by experience, to develop this subject, and impart to it the interest and attraction which it really merits.

We begin by saying that we must first have an accurate impression of the parts to be supplied by the vulcanite case. Accuracy here is all-important; for, however well the case may be constructed, a failure is inevitable, if the cast is incorrect. Hence, we take the impression in plaster, and aim to be careful and correct, in all our manipulations, that a satisfactory result may be attained. Having obtained a satisfactory impression, we allow it to stand and harden somewhat, then varnish it, and when the varnish has dried, oil and fill with plaster, so as to make the cast one-half or three-fourths of an inch thick, in the center, that there may be no surplus plaster. When the cast is hard to our liking, we detach it from the impression, trim, varnish and cover with tin foil' being careful to remove all the creases, by rubbing the surface of the tin with cotton-wool, saturated slightly with oil, and finishing the surface of the tin with a burnisher. We then mould on this cast a sheet of gutta percha for our model plate, using it to get our articulation, and as a base on which to set the teeth, as we grind and fit them. Some prefer and use wax plates; but they are not so satisfactory in our experience as gutta percha. We obtain our articulation as for gold work, and when the same is ready for use, we select, grind and arrange the teeth on our gutta percha plate, filling in, and moulding on wax, to suit our fancy, being careful to have a surplus, rather than a deficiency of the same, so as to permit sufficient scraping, cutting and finishing, to give the case a smoother face, when done. Whenever practicable, we use, and prefer the block teeth, as they are stronger, and look more natural than single teeth. We must not forget to bend the platinum pins, giving them a diverging direction from the blocks, or teeth, so as to amply secure, or dovetail them in the rubber. In joining the blocks or teeth, very careful grinding is necessary, else the appearance of the work will be greatly marred by the gum, which is certain to ooze through imperfect joints. This may be prevented by filling the joints with "Osteoplastic," which we have found more valuable for this purpose than any other agent as yet tested. Now we are ready to try the case in the mouth, when any existing imperfection can be remedied.

If all is right thus far, we are ready to flask the case, which, when properly set, is put in the heater, and gently warmed up, and then separated and the plate and wax removed. We then heat up, till pretty warm, the flask containing the teeth, whereby the wax will be absorbed by the investment, and the case prepared for the reception of the rubber. Previously to packing the same, we cut it in long and narrow strips, and such other shapes as will be most convenient for packing, and then lay them on a heated brick, covered with clean paper, so that the necessary plasticity will be imparted to the gum, to insure thorough packing. We should pack the case so full that there will be no fissures or defects in it when vulcanized, else the work may be so imperfect as to compel us to do our first works over. Having used as much gum as will fulfill the above indications, we then heat up the case holding the teeth, either in an oven or in the vulcanizer, till pretty warm, and then bring together the two flasks with a gentle pressure at first, and then heating again and pressing together again, alternately, till the flasks are closed tight together, when we are ready to submit the case to the action of the vulcanizer.

We should have remarked that before packing the rubber, grooves should be cut in the plaster for the escape of the surplus gum. To vulcanize, we fill our boiler with hot water till the case is covered, and then proceed to raise the steam till it reaches 330°, where we keep it for one hour, when the case will be generally as well vulcanized as if steamed for two hours and forty minutes, at 310°.

Our experience in constructing partial under sets, containing bicuspids and molars, has been rather limited, and so far, rather unsatisfactory. We find that the band passing behind the incisors, is liable to break, if reduced to such thinness as to remove the cumbrous feeling of fullness, and if left so thick as to exempt it from danger of breaking, the case becomes objectionable. Yet we doubt not that in efficient hands, this kind of case can be so made as to remove all objections.

In the construction of partial upper sets, we have uniformly succeeded, with little or no difficulty. In supplying the front teeth, we have found that, to prevent any unnatural fullness, the artificial gum should be in immediate apposition with the natural gum; and, to prevent the rubber from insinuating itself between the natural and artificial gum, we cut away all that part of the plaster cast lying immediately behind the artificial gum, and flow the plaster behind it, and under the anterior portion of the plate. By adopting this precaution, and pursuing the plan above recommended, of setting the teeth, there need be no more fullness than in mounting teeth on gold plate. But where fullness is necessary to restore the face to its original contour, then the rubber possesses claims superior to any other kind of work in general use. Indeed, we can conceive of no case where rubber may not be as successfully employed as any other material ; and in some cases, we do know that it is far superior to anything else. So far as we have the means of judging, it is agreeable, serviceable, and, therefore, satisfactory to the wearer, because it is *light*, *cleanly*, readily adapted, and economical, which, with a majority of our patients, is a desideratum.

These points conceded, and we can conceive of no good reason why they should not be, on a fair test, this style of work must, we predict, supersede, to a very great extent, all others now in vogue.

Repairing.—In this we have no experience, having, as yet, had no work to repair. But were a case of fracture, whether simple or compound, presented to us, we should have no fears about our ability to reduce it and restore the case to its wonted integrity. After giving the work as high a finish as possible, by using first our coarse files and cutters, following them with coarse, and then fine emery paper, and then finishing with brush and cotton wheels, we expose the case, in alcohol, to the action of the solar rays, by which the color is hightened, developed and perfected. This process may be continued with advantage for several days, provided time will permit.

When we come to "pass the Rubicon," by which we mean the insertion of the case in the patient's mouth, should it rock or ride on the hard ridge, we may secure a better adaptation by gently heating the case, and then bending it so as to fit more perfectly. This may be done with impunity; for, as soon as it cools, the case will be as inflexible as ever. If our work has been conducted throughout with care and skill, good results will follow, and all parties will be satisfied.

